# Figure-Ground Organization in Natural Scenes: Performance of a Recurrent Neural Model Compared with Neurons of Area V2

**DOI:** 10.1523/ENEURO.0479-18.2019

**Published:** 2019-06-25

**Authors:** Brian Hu, Rüdiger von der Heydt, Ernst Niebur

**Affiliations:** 1 Zanvyl Krieger Mind/Brain Institute, Johns Hopkins University, Baltimore, MD 21218; 2 Department of Biomedical Engineering, Johns Hopkins University, Baltimore, MD 21205; 3 Solomon Snyder Department of Neuroscience, Johns Hopkins University, Baltimore, MD 21205

**Keywords:** border ownership, natural scenes, neural networks, perceptual organization, recurrent processing

## Abstract

A crucial step in understanding visual input is its organization into meaningful components, in particular object contours and partially occluded background structures. This requires that all contours are assigned to either the foreground or the background (border ownership assignment). While earlier studies showed that neurons in primate extrastriate cortex signal border ownership for simple geometric shapes, recent studies show consistent border ownership coding also for complex natural scenes. In order to understand how the brain performs this task, we developed a biologically plausible recurrent neural network that is fully image computable. Our model uses local edge detector (B) cells and grouping (G) cells whose activity represents proto-objects based on the integration of local feature information. G cells send modulatory feedback connections to those B cells that caused their activation, making the B cells border ownership selective. We found close agreement between our model and neurophysiological results in terms of the timing of border ownership signals (BOSs) as well as the consistency of BOSs across scenes. We also benchmarked our model on the Berkeley Segmentation Dataset and achieved performance comparable to recent state-of-the-art computer vision approaches. Our proposed model provides insight into the cortical mechanisms of figure-ground organization.

## Significance Statement

Figure-ground organization is the process of segmenting an image into regions corresponding to objects and background. This process is reflected in the activity of cells in extrastriate cortex that show border ownership selectivity, encoding the location of an object relative to their receptive fields (RFs). We propose a model that can explain border ownership coding in natural scenes. Recurrent connections allow for integration of local and global object information, resulting in fast scene segmentation.

## Introduction

Figure-ground organization is critical for understanding the visual world around us. This process requires image segmentation, i.e., dividing the input image into regions corresponding to objects and background. Determining the correct assignment of each region border to its corresponding object is difficult due to clutter, occlusion, and the wide variety of features present in natural scenes. This problem has long fascinated researchers from psychology (Wertheimer, 1923; Koffka, 1935; Nakayama et al., 1995), neuroscience (Zhou et al., 2000; Craft et al., 2007), and computer vision (Sajda and Finkel, 1995; Ren et al., 2006; Teo et al., 2015; Wang and Yuille, 2016). Despite this long line of research, our understanding of the neural basis of figure-ground organization remains surprisingly limited.

**Figure 1.**
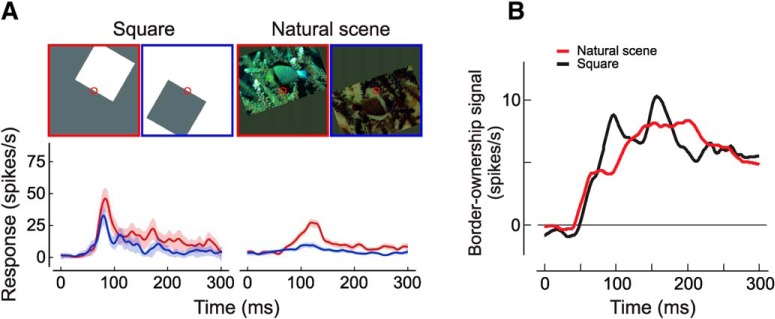
Consistency of border ownership coding. ***A***, Border ownership coding for an example cell. Upper panels, Red circles indicate the size and location of the cell’s RF. Visual stimulation within the RF is identical in the two presentations for the abstract figure (“square”), and nearly so for the natural scenes. This is achieved by rotating the object 180° about the RF and inverting color and luminance contrast of the image (Williford and von der Heydt, 2016). Stimuli with objects to the upper right of the cell’s RF (its “preferred” side) are outlined in red, while stimuli with objects to the lower left of the cell’s RF (“non-preferred” side) are outlined in blue. Lower panels, The cell’s peristimulus time histogram (PSTH) for the preferred side is shown by the red traces, while the PSTH for the non-preferred side is shown by the blue traces. The cell has a preference for objects located to the upper right of its RF on both the square and natural scene stimuli, as indicated by higher firing rates. Shading indicates 95% confidence intervals (note that shading is very narrow for the natural scenes data). ***B***, Population BOS. Across the entire population of recorded cells, the mean BOS (difference in firing rate between preferred and non-preferred sides) is similar for natural scenes (red trace) and for squares (black trace), suggesting a common, robust cortical grouping mechanism. Panels ***A***, ***B*** are modified from Figures 2 and 6, respectively, of Williford and von der Heydt (2016).

Zhou et al. (2000) first demonstrated that border ownership is implemented in the firing rates of individual neurons in extrastriate cortex. When the edge of an object is presented in the receptive field (RF) of one of these neurons, the cell responds with different firing rates depending on which side of its RF the object is located. A neuron’s difference in firing rates for when the object is located on the neuron’s preferred side versus when it is located on its non-preferred side is called the border ownership signal (BOS). Border ownership coding has been studied using a wide variety of artificial stimuli, including those in which the difference between foreground and background is defined by luminance (Zhou et al., 2000), motion (Von der Heydt et al., 2003), disparity (Qiu and von der Heydt, 2005), and transparency (Qiu and von der Heydt, 2007), as well as, more recently, by using natural stimuli such as faces (Hesse and Tsao, 2016; Ko and von der Heydt, 2018) and complex natural scenes (Williford and von der Heydt, 2016). A substantial fraction of neurons show consistent border ownership coding across natural scenes that matches their preference on artificial stimuli (Fig. 1*A*), with the timing of BOSs being similar for both types of stimuli (Fig. 1*B*).

How can cortical neurons modulate their activity based on visual input from locations at distances many times the size of their classical RFs? Proposed mechanisms based on asymmetric surround processing or lateral connections have difficulties explaining the relative timing of neuronal responses (see Comparison to other models). One class of models that does not suffer from this problem involves populations of grouping (G) cells which explicitly represent (in their firing rates) the perceptual organization of the visual scene (Craft et al., 2007; Mihalas et al., 2011; Layton et al., 2012). These cells are reciprocally connected to border ownership selective (B) cells through feedforward and feedback connections. The combined activation of grouping cells and cells signaling local features represents the presence of a “proto-object,” a term borrowed from the perception literature (Rensink, 2000). The use of proto-objects results in a structured perceptual organization of the scene. This proto-object-based approach, which we adopt here, is consistent with the results of psychophysical and neurophysiological studies (Duncan, 1984; Egly et al., 1994; Scholl, 2001; Kimchi et al., 2007; Qiu et al., 2007; Ho and Yeh, 2009; Poort et al., 2012).

However, with the exception of some computer-vision studies (Sakai et al., 2012; Teo et al., 2015), we are not aware of any models that have quantitatively tested border ownership selectivity on natural scenes. Russell et al. (2014) developed a model that is related to ours and that includes a class of border ownership selective cells, but that model is focused on the computation of saliency rather than the responses of BOS cells. Here, we propose a model based on recurrent connectivity that is able to explain border ownership coding in natural scenes. We compare our model results with experimental data and find good agreement both in the timing of the BOSs and in the consistency of border ownership coding across scenes. We also benchmarked our model on a standard contour detection and figure-ground assignment dataset, BSDS-500 (Martin et al., 2001) and achieve performance comparable to state-of-the-art computer vision approaches. Importantly, these machine learning techniques achieve their performance through extensive training using thousands of labeled images and very large numbers of free parameters, e.g., ≈10^8^ for VGGNet, a standard deep neural net model (Simonyan and Zisserman, 2014). In contrast, our model has less than ten free parameters and it requires no training whatsoever.

## Materials and Methods

### Model structure

Our approach is inspired by the proto-object-based model of saliency proposed by Russell et al. (2014), and it includes recurrent connections for figure-ground assignment, akin to the model from Craft et al. (2007). At the core of our model is a grouping mechanism which estimates figure-ground assignment in the input image using proto-objects of varying spatial scales and feature types (submodalities). These proto-objects provide a coarse organization of the image into regions corresponding to objects and background.

**Figure 2.**
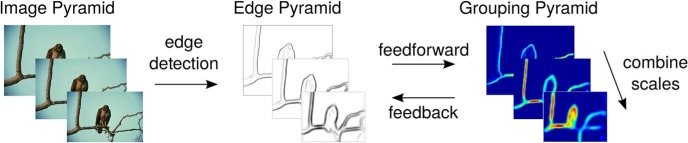
Overview of model computations. The image is edge filtered and then successively downsampled in half-octaves to create a pyramid of edge signal images (only three scales are shown). The same set of feedforward and feedback grouping operations is then applied at each level of the pyramid to achieve scale invariance. Feedback from grouping cells is combined across scales so that global context information can influence figure-ground segmentation. The model is run for a total of 10 iterations (one iteration includes one feedforward and one feedback pass through the model), and our final results are based on neural activity from the highest resolution scale of the image pyramid.

To achieve scale invariance, the algorithm successively downsamples the input image in steps of $\sqrt{2}$ to form an image pyramid spanning five octaves (Fig. 2). This is functionally equivalent to having similar RFs/operators at different spatial scales. The *k*–th level of the pyramid is denoted by using the superscript *k*. Unless explicitly stated, any operation applied to the pyramid is applied independently to each level and each feature type. Each layer of the network represents neural activity, which can be propagated from one layer to another via feedforward or feedback connections. We use a filter-based approach, where the RFs of neurons are described by filter kernels and the correlation operation (Eq. 3 below), is used to determine neuronal responses in a given layer from those in the previous layer. The model was implemented using MATLAB (MathWorks).

The first stage of the model extracts edges from the input image based on either luminance or color information (Fig. 2). We use the combination of RFs (CORF) operator, which is a model of V1 simple cells with push-pull inhibition (Azzopardi et al., 2014). We chose this operator due to its texture suppression properties, which can be beneficial when applied to natural images and because it is more biologically realistic than other computer vision algorithms. Our model does not require a specific edge detection method and could be modified to use other front-end edge detectors (e.g., Gabor filters). In the following, we only describe model computations on the luminance channel, but the exact same computations are also performed on the two-color channels (red-green and blue-yellow). As in Russell et al. (2014), the color channels were computed according to the methods outlined in the Itti et al. (1998) visual saliency model.

For a given scale *k*, the output of the edge detection stage of the model consists of simple (S) cells of eight different orientations *θ* and two contrast polarities, $S_{\theta ,L}^{k}(x,y)$ for light-dark edges *L* and $S_{\theta ,D}^{k}(x,y)$ dark-light edges *D*. For the two-color channels, the edge polarities are determined by color-opponent responses (e.g., red-green edges and green-red edges). Only the signal strength at the optimal orientation at each spatial location is used as input to the network. This simplification significantly reduces computation time by eliminating the calculation of responses for non-optimal orientations.

In contrast to previous approaches which combine simple cell responses into a contrast-invariant complex cell response (Russell et al., 2014), we keep the contrast-sensitive S cell responses available since they provide an informative cue for grouping along object edges. Objects tend to maintain similar contrast polarity along their boundaries, which may be useful for accurately determining figure-ground relationships. As a result, we have two sets of responses at each layer of our network corresponding to the two different types of contrast polarity, light/dark on the foreground/background border, and its opposite.

Next, for a given angle *θ*, each S cell feeds into an opposing pair of border ownership (B) cells. As a result, B cells are also sensitive to contrast polarity, as is the case for many experimentally observed border ownership receptive cells (Zhou et al., 2000). For each contrast polarity, we used one-to-one connections between S cells of one orientation and the corresponding pair of B cells. The two members of the pair have the same preferred orientation but opposing side-of-figure preferences.

**Figure 3.**
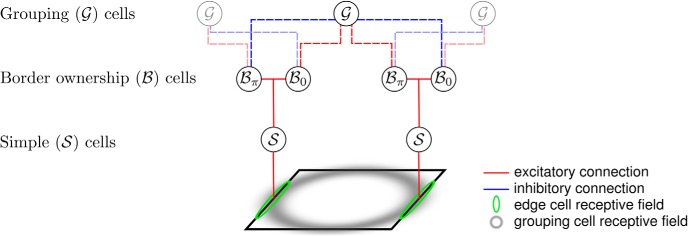
Structure of the recurrent neural network. Each circle represents a population of neurons with similar RFs and response properties. Red and blue lines represent excitatory and inhibitory projections, respectively. Solid and dashed lines represent purely feedforward and reciprocal feedforward/feedback connections, respectively. Edges and other local features of a figure (black square outline) activate simple cells (S) whose RFs are shown by green ellipses. S cells project to border ownership cells (B) that have the same preferred orientation and retinotopic position as the S cells they receive input from. For each location and preferred orientation, there are two B cell populations with opposite side-of-figure preferences. In the example shown, these are $B_{\pi }$, whose neurons respond preferentially when the foreground object is to the left of their RFs, and $B_{0}$, whose members prefer the foreground to the right side of their RFs. B cells have reciprocal, feedforward excitatory and feedback modulatory connections with grouping cells, G, which integrate global context information about objects. The RF of a G cell is shown by the gray annulus. It is also the projective field of this neuron for the modulatory feedback connections to B cells. Opposing B cells compete indirectly *via* feedback inhibition from G cells, which bias their activity and thus generate the BOS used to determine figure-ground assignment. The structure shown exists for both light objects on dark background [cell types $B_{\theta ,L}^{k}(x,y)$ and $G_{L}^{k}(x,y)$] and dark objects on light background [cell types $B_{\theta ,D}^{k}(x,y)$ and $G_{D}^{k}(x,y)$]. Grayed-out G cells represent proto-objects left and right of the one which is represented by the G cell in the center.

To infer whether the edges in $B_{\theta ,L}^{k}(x,y)$ and $B_{\theta ,D}^{k}(x,y)$ belong to figure or ground, knowledge of proto-objects in the scene is required. This context information is retrieved from a grouping mechanism (Fig. 3). Grouping cells (G) integrate information from B cells, and a given G cell responds to either light objects on dark backgrounds, $G_{L}^{k}(x,y)$, or dark objects on light backgrounds, $G_{D}^{k}(x,y)$. This computation is similar to the use of center-surround cells in the Russell et al. (2014) model. In contrast to their approach, our model does not require an additional class of center-surround cells, but instead allows G cells to directly integrate local feature information from B cells and then bias the activity of these same cells using reciprocal feedback connections. Our model runs in an iterative manner, with one iteration corresponding to one feedforward and one feedback pass through the model. G cell activity is combined across scales before each feedback pass, which allows the model to more accurately determine figure-ground assignment in a scale-invariant manner (Fig. 2).

A more detailed view of the structure of our model is shown in Figure 3. G cells integrate the B cell activity in a roughly annular fashion. This allows G cells to show preference for objects whose borders exhibit the Gestalt principles of continuity and proximity. G cell activity is defined by

1 $$G_{L}^{k}(x,y)=\left\lfloor \sum_{\theta }\;[B_{\theta ,L}^{k}(x,y)-B_{\theta +\pi ,L}^{k}(x,y)]*v_{\theta }(x,y)\right\rfloor$$

2 $$G_{D}^{k}(x,y)=\left\lfloor \sum_{\theta }\;[B_{\theta ,D}^{k}(x,y)-B_{\theta +\pi ,D}^{k}(x,y)]*v_{\theta }(x,y)\right\rfloor$$

where *θ* runs over all angles taken into account in the model (eight directed orientations, each with two side-of-figure preferences), ⌊·⌋ is half-wave rectification, and * is the correlation operator defined as

3 $$f(x,y)*g(x,y)=\sum_{m=-\infty }^{\infty }\sum_{n=-\infty }^{\infty }f(m,n)g(x+m,y+n)$$

The spatial structure of the G cell RFs is written in terms of the functions $v_{\theta }(x,y)$, defined as

4 $$v_{\theta }(x,y)=\frac{\exp\left[\left(\sqrt{x^{2}+y^{2}}-R_{0})\cos(\tan^{-1}(\frac{y}{x})-\theta +\frac{\pi }{2}\right)\right]}{2\pi I_{0}(\sqrt{x^{2}+y^{2}}-R_{0})}$$

where *θ* is the desired angle of the mask and the radius of the grouping cell RF *R*_0_ in this equation is set to two pixels. Because we rescale the input image at each level *k* of the image pyramid, the effective radius of each G cell RF $G^{k}(x,y)$ grows with the level of the pyramid, providing approximate scale invariance. The factor $\frac{\pi }{2}$ rotates the mask to ensure it is correctly aligned with the edge cells. *I*_0_ is the modified Bessel function of the first kind. We normalize each $v_{\theta }(x,y)$ by dividing by the maximum value over all positions (*x*, *y*). Conceptually, the G cell RF is a “donut” whose size is determined by the radius *R*_0_. We split this donut up into separate pieces according to the preferred orientations of the B cell neurons that project to the G cell.

Input to G cells is based on differences in preferred and non-preferred B cell activity (Eqs. 1, 2). This feedforward inhibition is not necessary for model convergence, but provides a means by which G cells can compete with each other via inhibition from B cells to G cells. In our simulation, the activity at the time of stimulus onset of each cell in a pair of B cells is numerically identical since both cells receive the same initial bottom-up input. As the difference in B cell activity is zero on the first iteration, we omit inhibition from non-preferred B cells and compute the activity of G cells based only on the preferred B cells on the first iteration. We also implement a simple form of local inhibition between the two complementary grouping pyramids, $G_{L}^{k}(x,y)$ and $G_{D}^{k}(x,y)$. The reason is that many objects are either dark on a lighter background or the inverse. Therefore, at each spatial location, only one type of G cell should be active, representing either a light or a dark object at that location. For each level of the pyramid *k*, we perform a winner-take-all value assignment,

5 $$G_{L}^{k}(x,y)\leftarrow \{\begin{array}{ll} G_{L}^{k}(x,y)\;\; & \text{if}\;G_{L}^{k}(x,y)>G_{D}^{k}(x,y)\\ 0\; & \text{otherwise} \end{array}$$

6 $$G_{D}^{k}(x,y)\leftarrow \{\begin{array}{ll} G_{D}^{k}(x,y)\;\; & \text{if}\;G_{D}^{k}(x,y)>G_{L}^{k}(x,y)\\ 0\; & \text{otherwise} \end{array}$$

Feedback from G cells to B cells is used to bias the responses of the B cells to correctly signal figure-ground assignment. The feedback depends on the contrast polarity of the G cell and the B cell. $B_{\theta ,L}^{k}$, the border ownership activity for a light object on a dark background is given by

7 $$\begin{array}{ll} B_{\theta ,L}^{k}(x,y)\;= & 2S_{\theta ,L}^{k}(x,y)\\ & \times \frac{1}{1+\exp(-(\underset{j\geq k}{\sum }\frac{1}{2^{j-k}}v_{\theta +\pi }(x,y)*G_{L}^{j}(x,y)-\underset{j\geq k}{\sum }\frac{1}{2^{j-k}}v_{\theta }(x,y)*G_{D}^{j}(x,y)))} \end{array}$$

and $B_{\theta ,D}^{k}$, the border ownership activity for a dark object on a light background is given by

8 $$\begin{array}{ll} B_{\theta ,D}^{k}(x,y)\;= & 2S_{\theta ,D}^{k}(x,y)\\ & \times \frac{1}{1+\exp(-(\underset{j\geq k}{\sum }\frac{1}{2^{j-k}}v_{\theta +\pi }(x,y)*G_{D}^{j}(x,y)-\underset{j\geq k}{\sum }\frac{1}{2^{j-k}}v_{\theta }(x,y)*G_{L}^{j}(x,y)))} \end{array}$$

where $v_{\theta }(x,y)$ is the kernel responsible for mapping object activity in the grouping pyramids back to the object edges (which is just the reciprocal kernel for the feedforward connections; Eq. 4), and the factor 2*^j^* ^-^*^k^* normalizes the *v_θ_*(*x*,*y*) operator across scales. Scales *j* greater than *k* in the equations above represent more global information. The model pools information across different spatial scales in a coarse-to-fine manner, with information from coarser scales first being upsampled to the resolution of the finer scale before being combined additively. The logistic function in the equations above enforces competition between B cells such that their total activity is always conserved, and each B cell has activity between zero and two times its bottom-up input activity, $S_{\theta }^{k}(x,y)$.

In the equations above, B cell activity is facilitated by G cell activity on its preferred side and suppressed by G cell activity on its non-preferred side. In other words, B cells receive (modulatory) facilitating feedback from G cells of the same contrast polarity on their preferred side and (modulatory) suppressive feedback from G cells of the opposite contrast polarity on their non-preferred side. This is motivated by neurophysiological results which show that image fragments placed within the extra-classical RF of a border ownership neuron can cause enhancement of the neuron’s activity when placed on its preferred side, and suppression if placed on the non-preferred side (Zhang and von der Heydt, 2010). Furthermore, modulating the scale-specific bottom-up S cell responses with G cell activity summed across spatial scales ensures that the B cell responses are scale-invariant. Neurophysiological results show border ownership coding for stimuli of varying sizes, with the latency of the BOS being essentially independent of the size of the figure (Zhou et al., 2000; Sugihara et al., 2011).

As discussed, figure-ground assignment occurs for both light objects on dark backgrounds and dark objects on light backgrounds. In our model, this is achieved by computing B cell activity independently for each contrast polarity and then summing the final steady-state activities for both the light and dark cell responses to give a final border ownership response independent of figure-ground contrast polarity. The B cell responses for light and dark objects can be combined giving a contrast polarity invariant result,

9 $$B_{\theta }^{k}(x,y)=B_{\theta ,L}^{k}(x,y)+B_{\theta ,D}^{k}(x,y)$$

While neurons with contrast-invariant border ownership responses are observed physiologically (Zhou et al., 2000), we do not implement them explicitly in our model for the sake of simplicity and computational efficiency. Their difference

10 $$B_{\theta }^{k}(x,y)-B_{\theta +\pi }^{k}(x,y)$$

is called the BOS by Zhou et al. (2000), a notation that we adopt. Its sign determines the direction of border ownership at pixel (*x*, *y*) and orientation *θ*, and its magnitude gives a confidence measure for the strength of border ownership.

Similarly, the G cell responses for light and dark objects are combined to a contrast polarity invariant result representing the presence of a proto-object of either polarity at location (*x*, *y*) and scale *k*:

11 $$G^{k}(x,y)=G_{L}^{k}(x,y)+G_{D}^{k}(x,y)$$

The output of the model is the G pyramid activity summed over all spatial scales and the differences in B cell activity at the highest spatial resolution, which provides a perceptual organization of the visual scene.

Objects can be perceptually segregated from each other or from the background because of differences in relative color or luminance. There are many other features underlying figure-ground segmentation, e.g., differences in texture, motion, etc. As mentioned previously, we use both luminance and color information from the image to perform the grouping operation. The same exact operations that were performed on the luminance channel are also performed on the two-color channels. We combine the final outputs of the B and G cells with an 80% weighting for the luminance channel and a 10% weighting each for the red-green and the blue-yellow color channels. Modifying the exact relative weighting does not qualitatively change our results.

### Code accessibility

The code/software described in this paper is freely available online at https://github.com/brianhhu/FG_RNN. The code is also available as Extended Data.

10.1523/ENEURO.0479-18.2019.ed1 Extended Data Download Extended Data, ZIP file.

### Model implementation

All simulations were performed on a 300-core CPU cluster running Rocks 6.2 (Sidewinder), a Linux distribution intended for high-performance computing. This allowed us to simultaneously run our model on multiple images, speeding up our testing time. We ran the model for a total of 10 iterations, with each iteration being one feedforward pass of B cell to G cell activity, followed by one feedback pass of G cell to B cell activity (Fig. 2). We generally found that the model converged after only a few iterations.

After convergence, the result is the self-consistent solution (fixed point) of the feedforward-feedback loop equation. Contour detection and figure-ground assignment results are computed from the population of B cells at the highest resolution level of the image pyramid, which has the same resolution as the input image. B cell activity is converted into a population vector code by summing the final activity across orientations, where the magnitude of the resulting vector at each pixel location represents the BOS (which we use as a measure of strength of contour detection, Model performance for contour detection and figure-ground assignment: comparison with standard benchmarks), and the direction of the vector provides a continuous figure-ground orientation label. For a given image, we normalize the BOS at each pixel (*x*, *y*) by its maximum value across the entire image, such that the BOS is bounded between –1 and 1. Negative BOS values indicate a predicted figure-ground orientation label which is opposite that of the ground-truth label.

### Comparison between model behavior and cell responses

To compare our model results with experimental results, we used a publicly available dataset of border ownership cell responses recorded during viewing of natural scenes by Williford and von der Heydt (2017), see the documentation of that dataset for more details about the stimuli, experimental design, and data analysis. Briefly, the dataset includes BOSs for each scene that was viewed by each recorded cell. Adopting the terminology of Williford and von der Heydt (2017), a “scene point” is a specific location in a specific image that is projected onto the RF of a cell. Scene points are selected such that they always lie on an object boundary. Note that an image can contain more than one scene point. In the following, we define consistency for the model or a given cell as the ratio of scene points with the same sign of BOS divided by the total number of tested scene points. For our analyses, we first selected a subset of cells (*N* = 13) from the population of recorded cells (*N* = 140) which had highly consistent border ownership responses, defined as having the same sign of border ownership on >80% of their tested scene points. To perform our analyses, we calculated the model’s BOS for the same set of scene points shown to the cells. We used a combination of different metrics to compare the BOS responses of one cell to that of another cell, or of one cell to the model, on the set of all common scene points viewed by both. Metrics used were cosine similarity, bootstrap and equivalence testing, and goodness of fit, which are explained below. The use of multiple metrics provides slightly different views of the model’s performance that is not biased by any one single metric. We found that the model’s performance was overall consistent across all measures that we used.

#### Cosine similarity

We characterize the behavior of a cell or the model by its BOS responses. When considering the correlation between responses of two cells, or a cell with the model, we first note that the Pearson correlation coefficient between the response vectors across scene points is not a suitable metric because it requires mean-centering the BOS responses. We therefore use an alternative measure of correlation between vector-valued functions that avoids this problem, the cosine similarity, which is commonly used in the field of natural language processing (Mihalcea et al., 2006), with some applications to neuroscience (Bruffaerts et al., 2013; Komorowski et al., 2013). For this method, all BOS responses of a given cell are described in terms of a single vector in a high-dimensional vector space where each (orthogonal) axis is the BOS response to one specific scene point. The component of the vector for one cell is the observed BOS for this dimension. The same applies for the comparison of a cell and the model.

For two arbitrary vectors *A* and *B* of equal dimensions, cosine similarity is defined as the scalar product of the two vectors normalized by the product of their lengths:

12 $$\cos(\theta )=\frac{A\cdot B}{\|A{\|}_{2}\|B{\|}_{2}}=\frac{\overset{n}{\underset{i=1}{\sum }}A_{i}B_{i}}{\sqrt{\overset{n}{\underset{i=1}{\sum }}A_{i}^{2}}\sqrt{\overset{n}{\underset{i=1}{\sum }}B_{i}^{2}}}$$

where *A_i_* and *B_i_* are the Cartesian components of the vectors *A* and *B*, respectively.

We can then compute the cosine similarity between any two vectors (e.g., between one cell and another cell or between a cell and the model) from Equation 12. It is bounded between –1 and 1, with the geometric interpretation that it measures the cosine of the angle between two vectors. Two vectors which are exactly the same will have a cosine similarity of 1, two vectors that are exactly opposite will have a cosine similarity of –1, and a cosine similarity of 0 indicates two vectors that are orthogonal or decorrelated.

To test the hypothesis that the model performs similarly to the most consistent cells from the experiment, we used bootstrap testing on the cell-cell and cell-model cosine similarities computed above. To perform the bootstrap test, means of the cell-cell and cell-model cosine similarities were calculated using resampling with replacement under the null hypothesis that the cell-cell and cell-model cosine similarities come from the same distribution. When computing means of cosine similarities, we used the Fisher *z*-transformation, which is a variance-stabilizing transformation for correlation coefficients. We calculated the bootstrap estimate of the difference in the means using a total of *N* = 10,000 samples.

#### Equivalence testing

Equivalence testing is a technique frequently used, for example, in the bioequivalence setting to determine whether the efficacy of a new drug or treatment is similar to that of an existing drug or treatment (Walker and Nowacki, 2011; Lakens, 2017). In standard hypothesis testing, the null hypothesis is that the means of two distributions are not different in a statistically significant manner. However, failure to reject the null hypothesis is not sufficient proof to conclude that the two distributions are actually similar, as the test may also fail due to not having enough statistical power (“absence of evidence is not evidence of absence”). In equivalence testing, the null hypothesis is, instead, that the means of the two distributions lie outside a pre-determined “zone of scientific indifference,” i.e., that they differ by more than the bounds of an interval within which two results are considered essentially equivalent. The alternative hypothesis (where the burden of proof lies) is that the means of the two distributions fall within this zone and can thus be considered equivalent. We consider the cell-cell and cell-model BOS values to be equivalent if the difference in their means falls with the interval [–0.25, 0.25], which is our zone of indifference. The equivalence test is performed by using two one-sided *t* tests from the Python *statsmodels* package.

#### Goodness of fit

We expressed goodness of fit by the coefficient of determination, which is defined as the fraction of total variance explained by the model (Holdgraf et al., 2017). Because neural BOS and model BOS have different scales, we added a scale factor to the model that was determined for each cell by a least-squares fit.

Each cell’s response contains a repeatable component $\sigma_{response}^{2}$ which is the same in response to the same stimulus and which we attempt to capture with our model in the variable $\sigma_{predicted}^{2}$, and a noise component, $\sigma_{noise}^{2}$. The latter is random and its contribution can be estimated from the responses to repeated presentations of the same stimuli. Because our model is deterministic, it is unable to capture the noise component present in the cell responses. We only care about the explainable variance, which is the total response variance minus the noise variance. As a result, we define our goodness of fit measure by computing the fraction of explainable variance that is actually explained by the model,

13 $$R^{2}=\frac{\left[\sigma_{predicted}^{2}-(1/N_{s})\sigma_{noise}^{2}\right]}{\left[\sigma_{response}^{2}-\sigma_{noise}^{2}\right]}$$

where we apply a correction term in the numerator for the fraction of the noise variance captured by fitting a scale factor. This is determined by the ratio of the degrees of freedom in the least-squares fit (1 for the scale factor) and the degrees of freedom in the data (the number of scene points, *N_s_*; see DiCarlo et al., 1998; Wu et al., 2006). Because the noise variance is estimated from the data, the computed model goodness of fit may contain small errors. Therefore, we also report average values over the population of cells. Our statistical analyses are summarized in Table 1.

**Table 1.** Statistical analysis

Line	Data structure	Type of test	Power
a	Approximately normal	Bootstrap	*p* = 0.11
b	Approximately normal	Equivalence test	*p* = 0.03
c	Normal	Significance of correlation coefficient	*p* < 0.5

## Results

### Model performance for contour detection and figure-ground assignment: comparison with standard benchmarks

We benchmarked our model on the publicly available Berkeley Segmentation Dataset, BSDS-500 (Martin et al., 2001). We did this in the context of two tasks: contour detection and figure-ground assignment. For the contour detection results, we report F-scores, the harmonic means of precision and recall, averaged over all test images. Precision is the fraction of boundary pixels detected by the model that are true boundary pixels (i.e., those marked by humans). Recall is the fraction of true boundary pixels detected by the model. The F-score provides a summary score that captures the trade-off between the accuracy and noise of contour detection. For the figure-ground assignment results, we report mean accuracy (percentage of correctly labeled figure-ground edges) averaged over all test images. We used publicly accessible benchmarking code made available by the authors of the original papers for contour detection (Arbeláez et al., 2011) and figure-ground assignment (http://users.umiacs.umd.edu/~cteo/BOWN_SRF/) to do our analysis and comparisons with other approaches. We report our results on the contour detection and figure-ground assignment tasks in Tables 2, 3, respectively.

**Table 2.** Contour-detection results on the BSDS-500 dataset

	Contour		
	ODS	OIS	AP
Human	0.80	0.80	-
Our approach	0.64	0.65	0.51
gPb-owt-ucm	0.73	**0.76**	0.73
SE	0.73	0.75	**0.77**
SRF	0.73	0.74	0.76

Numbers shown are the F scores when choosing the optimal scale for the entire dataset (ODS) or per image (OIS), as well as the AP. Average agreement between human subjects is captured by the “human” scores, which provides an upper bound on model performance. In this table and in Table 3, an absolute performance maximum by an algorithm is indicated by boldface numbers.

**Table 3.** Figure-ground assignment results

	Figure-ground
	Mean accuracy
Human	83.9%
Our approach	71.5%
SRF	**74.7%**
Global-CRF	68.9%
2.1D-CRF	69.1%

Numbers shown are the mean accuracy across all matched scene points.

**Figure 4.**
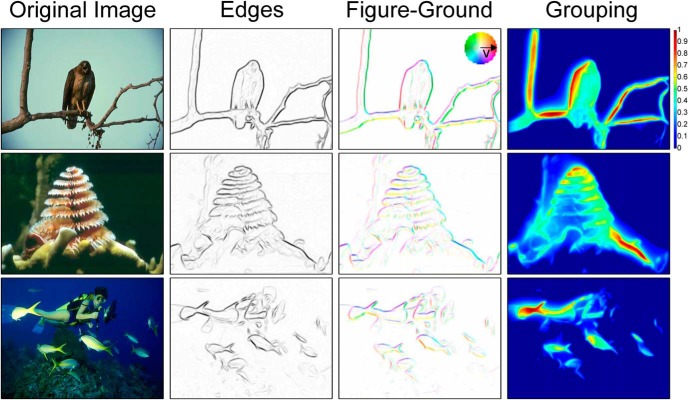
Example results of our model on images from the Berkeley Segmentation Dataset. Columns from left to right are the original images, the edge activity, the border ownership cell activity (representing figure-ground assignment), and grouping cell activity. For the figure-ground assignment, each edge is represented by a hue and a saturation value (see color wheel inset). The hue of the edge represents the figure-ground orientation label with the arrow convention shown in the color wheel (e.g., red represents an object located to the right) and the saturation of the edge represents the strength of the BOS. Grouping cell activity is color coded and normalized, with warmer colors representing higher activity (see color bar at right).

Importantly, parameters were not tuned separately for the two tasks: our model uses the same set of parameters for both contour detection and figure-ground assignment. Examples of our model output are shown in Figure 4. We show the original input image, the edge maps, the BOSs, and the final grouping maps. Although we did not specifically design our model to achieve good performance on the contour detection task, we hypothesized that BOS is a good correlate of the perceptual saliency of object contours. As such, we use the strength of the BOS (absolute value, independent of figure-ground orientation) as the model output for the contour detection task.

We compare our model to three state-of-the art approaches from the computer vision field: ultrametric contour maps (gPb-owt-ucm; Arbeláez et al., 2011), structured edges (SE; Dollár and Zitnick, 2015), and structured random forests (SRFs; Teo et al., 2015). We quantify performance for the contour detection task using three different measures: the best F-score on the dataset for a fixed scale (ODS), the average F-score on the dataset using the best scale for each image (OIS), and the average precision (AP), which is the area under the precision-recall curve. We refer the reader to Arbeláez et al. (2011) for a more in-depth discussion of these metrics. Overall, we achieved an F-score of 0.64 on the contour-detection task when evaluating using the optimal dataset scale. Our F-score improves slightly (to 0.65) when evaluating using the optimal image scale. We achieve lower AP (0.51) compared to the other models due to the lower recall range of our model, which may be the result of limitations in the initial edge detection method we used. All three cited models achieve F-scores of 0.73 using the optimal dataset scale (Table 2). Again, we emphasize that we did not design our model for the contour detection task, but we were nevertheless able to use computed BOSs from the model as a measure of contour detection strength.

For the figure-ground assignment task, we quantify our results using the mean accuracy of figure-ground assignment across all labeled contours in the test images. The model’s figure-ground label for a given scene point in the image is considered correct if it falls within ±90° of the true (i.e., human-defined) figure-ground label. We compared our model to SRFs (Teo et al., 2015) and two conditional random field approaches, Global-CRF (Ren et al., 2006) and 2.1D-CRF (Leichter and Lindenbaum, 2009). SRFs achieved a mean accuracy of 74.7%, exceeding that of the two other conditional random fields approaches (Ren et al., 2006; Leichter and Lindenbaum, 2009) which were below 70%. Surprisingly, despite the lack of training, our model outperforms these latter models with a mean accuracy of 71.5% (Table 3). There is also a recent deep learning approach to the same problem (Wang and Yuille, 2016), but since the results of this method were not benchmarked using the standard tests employed by the other methods, we did not include them in our comparison.

In summary, we find that some current computer vision approaches are able to achieve better performance than our model based on the evaluation metrics described above, but they require extensive training, i.e., tuning of a large number of parameters using large sets of training data. In contrast, our model is built based on first principles and does not require any specific form of training. Although our model is outperformed by some state-of-the-art methods, it does represent an alternative approach based on biologically plausible neural computations that require very little training or tuning of parameters. It therefore may add substantial insight into the underlying mechanisms involved in solving these tasks which is not readily available through solutions that rely on extensive training.

### Timing of the BOS

**Figure 5.**
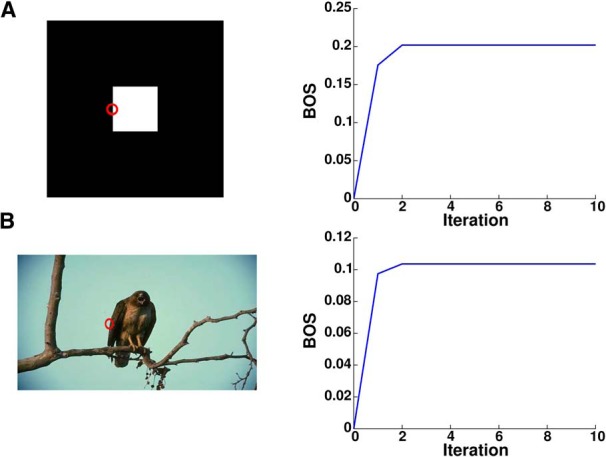
Time course of border ownership coding in the computational model, which achieves correct border ownership assignment within two to three iterations. The RF of one model border ownership cell is shown by the red circle. The input image and time course of this BOS cell are shown for both standard square stimuli commonly used in experiments (***A***) and an example scene from the Berkeley Segmentation Dataset (***B***).

We tested our model on the standard square stimuli used to determine border ownership preference in experiments (Zhou et al., 2000), as well as a wide array of natural scenes from the Berkeley Segmentation Dataset. We found that our model converges within a few iterations, typically two to three, demonstrating that only a few feedforward and feedback passes are needed to determine figure-ground assignment for a given image (Fig. 5). Given that white-matter projections in the brain are quite fast, we assume that a single feedforward and feedback pass in our model takes ∼10 ms. As the model converges within two to three iterations, the BOS will reach its peak within 20–30 ms of the initial visual response. A similar time course has been observed in the experimental data, with the BOS appearing ∼30 ms after visual response onset (Zhou et al., 2000; Williford and von der Heydt, 2016). The similar time course of BOS tuning on both artificial and natural stimuli suggests a common cortical mechanism for grouping, which is also supported by previous experimental results demonstrating consistent border ownership coding across these different types of stimuli. Our model is able to reproduce this result, showing a similar time course for border ownership coding on both the square and natural scene stimuli.

### Model performance on border ownership coding: comparison with experimental results

**Figure 6.**
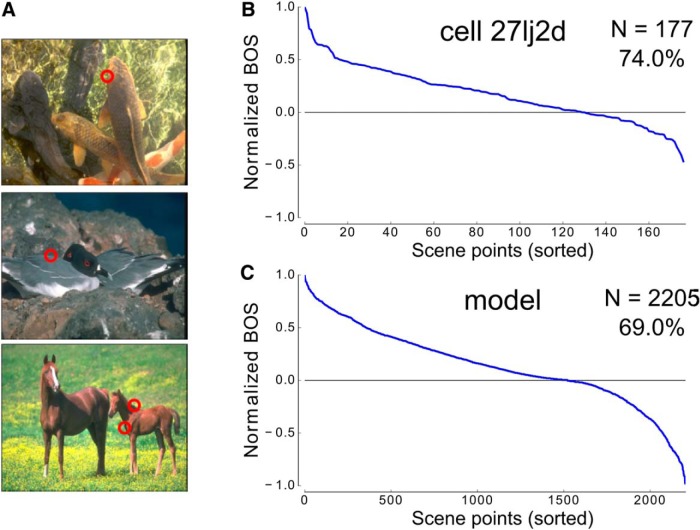
Cell and model consistency across scene points. ***A***, Examples of scene points that were used to test border ownership selectivity during the experiments. Red circles represent scene points within the images, which were centered on the RFs of border ownership selective neurons during the experiments and during our testing of the model. A single image could contain multiple scene points, as shown by the example in the bottom row. ***B***, The normalized BOS for example cell 27l*j*2*d* is shown according to each scene point, with scene points sorted in decreasing order by strength of BOS for this cell. The cell achieved a consistency of 74.0% across all tested scene points (*N* = 177). ***C***, The normalized BOS for the model is shown with the same convention as in ***B***, with scene points sorted by strength of model BOS. The model achieved an overall consistency of 69.0% across all tested scene points (*N* = 2205).

The model exhibits consistent border ownership coding across a large number of natural scenes, similar to the most consistent cells (consistency being defined in Comparison between model behavior and cell responses) from the experiment. Figure 6 compares the BOSs sorted in descending order by scene point for an example cell (Fig. 6*B*) and for the model (Fig. 6*C*). We chose this cell because it was tested with 177 scene points, the largest number for any single cell in the dataset. It showed a consistency of 74.0%. A large number of cells in the dataset were highly consistent, even more so than the cell illustrated in Figure 6, including 13 cells with >80% consistency. Within this subset of cells, three cells exceeded 90% consistency. In comparison, the model showed an overall consistency of 69.0% across 2205 tested scene points (the full set of scene points viewed collectively by any of the highly consistent cells). Although the model was tested with more than an order of magnitude more scene points than the example cell in Figure 6, it still remained highly consistent. This level of consistency is similar to the ∼70% accuracy the model achieved on the figure-ground assignment benchmark.

**Figure 7.**
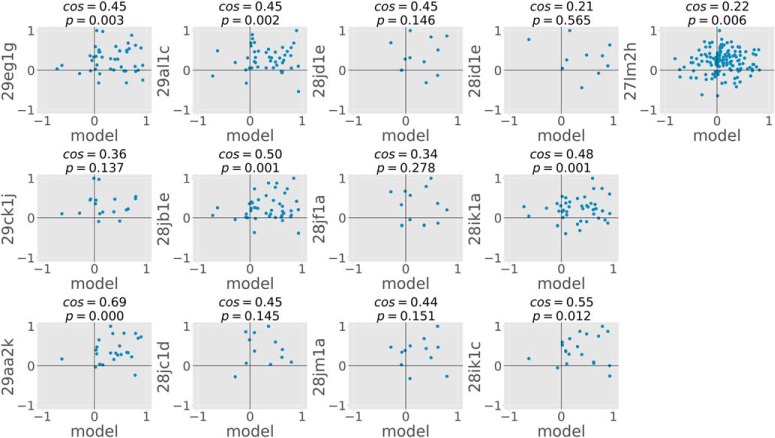
BOSs of each of the highly consistent cells (*N* = 13) plotted against BOS of the model. Each subplot shows a scatter plot of one cell’s normalized BOS against the model’s normalized BOS on the common set of scene points viewed by both. Each dot corresponds to one scene point. Note that all data points in the upper-right and lower-left quadrants indicate agreement of model and cell behavior while data points in the other two quadrants indicate disagreement. The cosine similarity metric along with the associated *p* values (test whether the cosine similarity metric is different from zero) are shown above each scatter plot. Cosine similarities for the cell-model comparisons ranged from 0.21 to 0.69, with 7/13 cells having cosine similarities that were significantly different from zero.

We also used the cosine similarity metric (see Cosine similarity) to quantify similarity in BOS responses between cells and similarity between cells and the model on a shared set of scene points. Despite the large diversity in cells and their responses, we found that our model was able to largely explain the border ownership coding of highly consistent cells on natural scenes. Figure 7 shows the comparison of cosine similarities between model and cells on a per-cell basis for all 13 highly consistent cells. The model-cell cosine similarities were all positive, ranging from 0.21 to 0.69, with a mean similarity of 0.44. Given biological noise and inter-cell differences, it is impossible that the model-cell cosine similarities reach unity. To characterize an upper bound on the cosine similarity values, we also calculated the cosine similarities between all pairs of highly consistent cells (13 cells, *N* = 58 pairs). For the cell-cell comparisons, the cosine similarities ranged from 0.14 to 0.91, with a mean similarity of 0.54. Bootstrap testing revealed no significant statistical difference between the means of the cell-cell and cell-model cosine similarities (*p* = 0.11).

Since the absence of statistically significant difference between two distributions by itself is not evidence that they are the same, we used equivalence testing (see Equivalence testing) on the means of the cell-cell cosine similarities and model-cell cosine similarities. In contrast to standard hypothesis testing, in equivalence testing the null hypothesis is that a significant difference between the two population means does exist. Our results revealed no significant difference between the cell-cell and model-cell cosine similarity values based on a zone of scientific indifference of [–0.25, 0.25], leading us to reject the null hypothesis (*p* = 0.03). We conclude that the performance of our model is indistinguishable from that of the set of highly consistent cells in the dataset.

**Figure 8.**
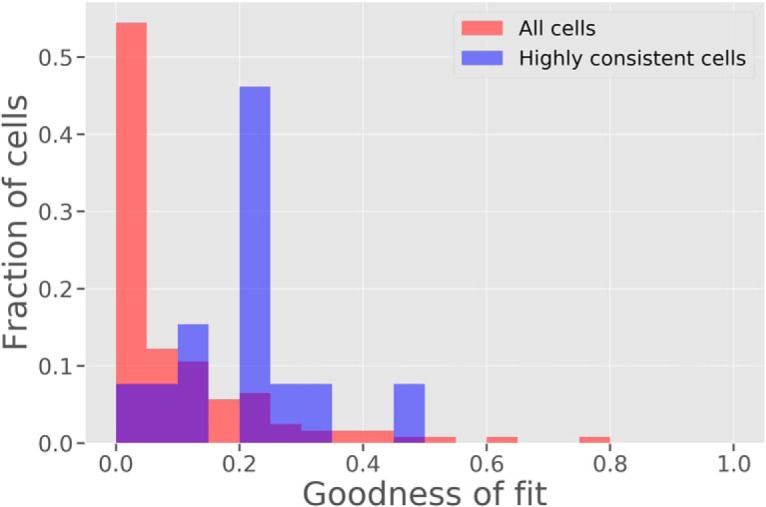
Model goodness of fit to the BOSs across images of all cells in the dataset (red) and to only the highly consistent cells in the dataset (blue). There was a total of 140 cells and 13 highly consistent cells. The model is able to better predict the BOSs of highly consistent cells, with a mean goodness of fit value of 0.21 compared to 0.08 for all cells in the dataset.

We also computed linear regression fits between the cell BOS responses and the model BOS responses on a per-cell basis. Each regression results in an *R* ^2^ goodness of fit value (Eq. 13), which gives a measure of the percentage of variance that the model is able to explain. The noise variance for each cell was estimated from the responses of the cell to separate presentations of the identical scene point and averaged over all scene points presented. The *R* ^2^ goodness of fit values for the highly consistent cells ranged from 0.05 to 0.55, with a mean value of 0.24. For two of the 13 highly consistent cells, the *R* ^2^ values exceeded 0.3, indicating that the model was able to capture >30% of the explainable variance. When we computed the *R* ^2^ goodness of fit values over all cells, the mean value was 0.14. Figure 8 shows a histogram of the goodness of fit values over the entire dataset. This shows that the model was better able to predict the responses of the highly consistent cells. The fact that the fraction of the variance explained by the model is low when cells with low consistency are included is not surprising because low consistency across scene points indicates that these cells are not primarily concerned with computing figure-ground relationships. Single-cell recording studies like the one by Williford and von der Heydt essentially pick cells at random, and the visual cortex contains different populations of cells performing a variety of computations in parallel.

## Discussion

### Understanding the cortical mechanisms of figure-ground organization

We propose that a simple grouping mechanism can explain figure-ground organization in natural scenes. Grouping cells in our model have annular RFs, which implement Gestalt principles like convexity, continuity, and proximity. Importantly, the design of these RFs was based on first principles, and not due to any training or parameter tuning on natural scenes, as is common in machine learning approaches. We show that this RF structure is useful for assigning figure-ground relationships on both artificial and natural stimuli. These RFs capture the convex shape of objects, which has been shown to be an important cue from the analysis of natural scene statistics (Sigman et al., 2001). Our model does not use higher-level object identity information, which may influence segmentation based on object familiarity. While such information likely is used in certain situations, the fast time course of border ownership assignment in extrastriate cortex makes it unlikely that these signals are informed by cortical object recognition modules, like those found in inferotemporal cortex where response latencies are considerably longer. Instead, we propose that the grouping mechanisms in our model operate at intermediate levels of the visual hierarchy to structure the visual scene into proto-objects useful for further visual processing.

Our model border ownership responses show close agreement with the responses of highly consistent cells from the Williford and von der Heydt (2016) experiments. This is surprising given the diversity of cell responses to different natural scenes, even highly consistent cells themselves are not entirely consistent with each other, perhaps indicating that a population of neurons is needed to accurately encode figure-ground relationships (Hesse and Tsao, 2016). However, our model, which is based on the simple principle of an annular grouping cell RF, is able to capture the responses of many of these neurons.

The model relies on feedforward and feedback connections via fast white-matter projections between visual areas. This is consistent with the rapid appearance of BOSs after visual stimulus onset. This is a clear difference between our model and others which rely either on feedforward or on lateral connections. Our model makes testable predictions about the role of feedback in figure-ground segmentation. One experimental prediction is that disrupting feedback from higher visual areas (specifically, the feedback from grouping cells) would impair the figure-ground assignment process, and potentially result in poor border ownership assignment and segmentation of objects in the scene. Models based purely on feedforward processing do not make this prediction. We also predict the existence of contrast-sensitive and color-sensitive grouping cells, which send reciprocal feedback connections to similarly-tuned border ownership cells. This is a prediction awaiting experimental testing.

We also use a variety of grouping cells of different scales, which allows our model to achieve relative scale invariance across the range of object sizes present in natural scenes. The main contribution of our present work is the development of a fully-image computable model of figure-ground organization that can be applied to natural scenes. Our model provides a quantitative means to study the potential cortical mechanisms of this process, including the relative contribution of feedforward and feedback processing.

### Comparison to other models

A number of computational models have been developed to explain border ownership selectivity. One model class assumes that border ownership coding is achieved purely by feedforward mechanisms, such as the asymmetric organization of surrounds (Nishimura and Sakai, 2004, 2005; Sakai et al., 2012) or global surround inhibition (Supèr et al., 2010). Pure feedforward models predict similar latencies of the BOS regardless of the stimulus, but recent results show that border ownership assignment of stimuli with illusory contours is delayed by ~30 ms compared to full stimuli (Hesse and Tsao, 2016).

Other models propose propagation of neural activity along horizontal connections within early visual areas using a diffusion-like process (Grossberg, 1994; Sajda and Finkel, 1995; Pao et al., 1999; Kikuchi and Akashi, 2001; Baek and Sajda, 2005; Zhaoping, 2005; Zucker, 2012). Like the feedforward paradigms, these models have difficulties explaining the exact timing of neuronal signals. Zhou et al. (2000) showed that the BOS appears as soon as ≈25 ms after the first response to the stimulus. Propagation along horizontal fibers over the distances used in the experiments would imply a delay of at least ≈70 ms (based on the conduction velocity of horizontal fibers in primate V1 cortex from Girard et al. (2001), we are not aware of corresponding data for V2). Such models are also difficult to reconcile with the observation that the time course of border ownership coding is largely independent of figure size (Sugihara et al., 2011). Furthermore, these models (as well as others, Layton et al., 2012) are largely untested on natural stimuli, and it remains to be seen if previous results on artificial stimuli will generalize to more difficult real-world conditions.

The only other models that we are aware of that have been tested on natural stimuli either used locally computed cues (Fowlkes et al., 2007) or feedforward processing to determine figure-ground assignment (Nishimura and Sakai, 2005; Sakai et al., 2012; Russell et al., 2014). The Fowlkes et al. (2007) model required human-labeled image contours as input, and operated only on local boundary information from image patches but did not incorporate luminance or color information. The Russell et al. (2014) model is conceptually similar to ours, involving similar classes of grouping and border ownership neurons. However, their model is purely feedforward and involves an additional class of center-surround neurons which are needed to generate a coarse segmentation of the image. Furthermore, Russell et al. (2014) did not quantitatively study border ownership in their model, instead focusing on applications to visual saliency. The Sakai et al. (2012) model is also a purely feedforward model which determines figure-ground relationships based on asymmetric surround contrast. Different from our model, their approach was not fully image-computable. Instead, Sakai et al. (2012) tested model performance on human-labeled contours from the Berkeley Segmentation Dataset. In addition, their model was only applied to luminance information and ignored color information, so all input images were first converted to grayscale. Our model is fully image-computable, which means that it can be applied to any image, including those without human-labeled contours. Our model is also able to incorporate both luminance and color information from images, which will allow for future study of the relative contributions of these two cues on grouping.

Our model is a member of a broad class of theoretical models that achieve image understanding through bottom-up and top-down recurrent processing (Ullman, 1984; Hochstein and Ahissar, 2002; Roelfsema, 2006; Epshtein et al., 2008). Our model is explicit in that feedback connections from higher visual areas modulate the responses of early feature-selective neurons involved in the related processes of contour detection and figure-ground segmentation. Despite requiring feedforward and feedback passes of information through the model, our model converges quickly, consistent with the fast establishment of figure-ground assignment in the visual cortex.

Experimental results also suggest that feedback from higher visual areas may be useful for tasks such as contour tracing (Roelfsema et al., 1998) and segmentation of texture-defined figures (Lamme, 1995). As in our approach, computational models of these processes involve a hierarchy of visual areas that are recurrently connected (Poort et al., 2012). While our model deals primarily with the segmentation of contour-defined objects, grouping of the surfaces that belong to objects and the filling-in of these surfaces from contour information remains an active area of research.

As mentioned above in Model structure, where we defined the structure of the model, the purpose of our study is to demonstrate how neuronal circuitry can integrate information from different classes of features to achieve perceptual organization. For this reason, we combined a small number of different features (contrast in intensity and two-color opposites). Nevertheless, there are obviously many other cues used by the visual system to set apart objects from each other and from the background, e.g., texture contrast, stereo/disparity, motion, etc. In addition to these context-defined cues, local information likely plays a role, e.g., the presence of L, X, and T junctions. Craft et al. (2007) showed that such local information (using the example of T junctions) can be incorporated into a recurrent network that has an overall structure similar to ours (although their model works on highly abstracted input information and is not image computable).

Another class of available information is based on differences in image statistics on the two sides of the border. These differences can be quantified in the spectral domain and they contribute significantly to figure-ground segmentation in natural scenes (Palmer and Ghose, 2008; Ramenahalli et al., 2014). Although Williford and von der Heydt (2016) did not find an influence of local edge structure on the border ownership responses in nonhuman primate visual cortex, the edge profile is known to be used by humans to distinguish foreground from background (Von der Heydt and Pierson, 2006; Palmer and Ghose, 2008). The parallel architecture of our model (as well as that of the primate visual system) makes it easy to add these additional channels, as well as others, to the existing three channels (intensity, red-green, blue-yellow). This remains the topic of future work.

One criticism addressed at many computational models is that they are “tailor-made” to explain one particular phenomenon. While their performance may be impressive in this regard, it is clear that a biological nervous system needs to cope with more than one task. The model we are presenting in this study is designed to primarily explain border ownership coding, the phenomenon for which we have quantitative neurophysiological data. The model, indeed, explains these data quite convincingly. In addition, as we have shown in Model performance for contour detection and figure-ground assignment: comparison with standard benchmarks, the model’s performance is also competitive with state-of-the-art computational models that have been specifically designed for two different standardized tasks: contour detection and figure-ground assignment in a benchmark data set of natural scenes. We find it very encouraging that our simple model with a minimal number of tuned parameters (many orders of magnitude less than standard machine-learning algorithms) can explain several intermediate-vision processes simultaneously.

### Grouping neurons

There is as yet no direct neurophysiological evidence for grouping neurons, although previous studies have found neurons in V4 that respond to contour segments of various curvatures (Gallant et al., 1996; Pasupathy and Connor, 2002; Brincat and Connor, 2004). Our choice of an annular, donut-shaped grouping cell kernel is a simplification which, prima facie, seems ill-suited to represent objects like thin, elongated shapes or concave shapes. A standard representation of complex shapes in computer vision is the medial axis transform which can generate a skeleton-type abstraction of any shape (Blum, 1967; Hung et al., 2012). Previous work has shown that the population activity of grouping cells is a close approximation of the medial axis transform (Ardila et al., 2012) and thus can represent any arbitrary shape. Furthermore, although we do not make use of the population activity in this study, in practice we find that the combination of scale invariance and recurrent processing allows the model to accurately predict figure-ground relationships in natural scenes. We also do not rule out the possibility that other types of grouping neurons may also exist, including those that respond to straight contours (Hu and Niebur, 2017), gratings (Hegdé and Van Essen, 2007), illusory surfaces (Cox et al., 2013), or 3D surfaces (He and Nakayama, 1995; Hu et al., 2015). For the sake of simplicity in this proof-of-concept study, we do not attempt to model the whole array of grouping neurons that may exist.

Furthermore, there is indirect evidence showing the potential influence of grouping cells on the spike timing of border ownership selective neurons in extrastriate cortex. Martin and von der Heydt (2015) showed that action potentials of border ownership selective neurons that represent the same object are more synchronized than those neurons that represent different objects (see also Dong et al., 2008). This is exactly what is expected if the former group of cells receives common input from grouping cells that represent one object while neurons coding for different objects receive input from different grouping cells that fire independently.

Grouping neurons may also interact with higher-level object recognition centers, such as inferotemporal cortex, as familiarity with certain objects such as faces may influence figure-ground assignment. This is currently an area of active research (Ko and von der Heydt, 2018). Furthermore, grouping neurons may be multi-modal, in that they respond to many different features that may aid the scene segmentation process, such as disparity, motion, etc. In fact, experimental results show that border ownership selective neurons have consistent border ownership tuning across 2D luminance and 3D disparity cues (Qiu et al., 2005). We have not yet incorporated these additional features into our model, but this represents a potential area of future research.

### Scope and limitations of the model

Our model assigns distinct roles to the different visual areas, e.g., edge processing in V1 by simple cells, figure-ground assignment in V2 by border ownership selective cells, and grouping of proto-objects, possibly in V4. Neurons in these different areas have additional ranges of selectivity than the ones we assign them in our model. Our model also produces a rough approximation of the time course of border ownership coding through a rate-based, iterative process. As such, it does not allow us to study the dynamics of the recurrent network at a finer timescale. For example, the attention-dependent modulation of spike-spike synchrony between border ownership neurons that are part of the same object is of particular interest (Martin and von der Heydt, 2015; Wagatsuma et al., 2016). Furthermore, we focused more closely on the border ownership cell activity in our model and did not specifically study the grouping cell responses of our model, but the combined activity of grouping cells across scales could be used to study a wide range of other visual phenomena, including object segmentation and visual saliency.

## Synthesis

Reviewing Editor: Li Li, New York University Shanghai Decisions are customarily a result of the Reviewing Editor and the peer reviewers coming together and discussing their recommendations until a consensus is reached. When revisions are invited, a fact-based synthesis statement explaining their decision and outlining what is needed to prepare a revision will be listed below. The following reviewer(s) agreed to reveal their identity: Xin Huang.

Both reviewers agreed that the ms addressed an interesting question in the field and proposed a biologically plausible model that can help understand the underlying neural mechanism. Both also raise some minor concerns that should be addressed before the ms can be considered for publication. I summarize their review below.

Reviewer 1:

This is a relatively good study. Authors describe a model for this border assignment coding process. This model is based on a recurrent neural network in which two groups of cells interact to integrate local edge and grouping features in a modulatory feedback approach. This model somehow supports the experimental findings and showed a acceptable performance. This study can provide insights to cortical mechanisms of object-ground organization. There are some suggestions

1. in the manuscript, in the methods and results section please cite the references referred by authors. for instance the references used for descriptions of the metrics used in this study (2.4.1 and 2.4.2 and 2.4.3)

2. In the comparisons between the model and the present dataset, linear regression used for goodness of fit is a good tool? as this is a linear model but the nature ofthe proposed mode land the properties of the neural functions is nonlinear. It would be good if authors discuss the reliability of the used metrics.

Reviewer 2:

In this study, the authors developed a biologically plausible neural network model to understand how neurons in primate extrastriate cortex signal border ownership for complex natural scenes. The model consists of simple (S) orientation-selective cells, border ownership (B) cells, and grouping (G) cells. The key idea of the model is that G cells integrate local feature information from B cells and bias the activity of B cells via recurrent feedforward-feedback connections, making B cells border ownership selective. The authors compared their model results with published neurophysiological data of cells' border ownership responses to natural scenes and found good agreement. The authors also benchmarked their model on the Berkeley Segmentation Data Set. Determining which object owns a border in complex natural scenes is a difficult problem. This study builds on the previous modeling works involving recurrent connections (Craft et al., 2007) and proto-object based approach (Russel et al., 2014). The current study demonstrates that a grouping mechanism that estimates figure-ground assignment using proto-objects of varying spatial scales and feature types provides a simple and elegant solution for assigning objet borders in natural scenes. The authors conducted the study rigorously. The manuscript is overall well written. I think this study represents an important step forward in the field and the results are significant. I have several relatively minor comments/suggestions.

1. Line 171 - 179: “It has been known for a long time that feedfoward (thalamocortical) input is characterized by direct, monosynaptic, excitation followed by cortically mediated disynaptic inhibition. ... These intra-cortical interactions break the symmetry of bottom-up inputs to the B-cells. While we do not model the details of local (intracortical) processing, we implement the delay of the arrival of inhibitory influences by omitting all inhibition between B cells on the first iteration when computing the activity of the G cells.”

It is unclear to me what is implemented about the delay of the arrival of inhibitory influences. Do the authors simply mean that there is no intracortical inhibition between B cells in the model, but there can be intracortical inhibition via feedback connection from G cells, following the first iteration? This can be clarified.

2. Pooling across different spatial scales seems to be important for the success of the model, but the description of this mechanism is not very clear.

Line 194, 2-j. Should it be 2j-k ? What is the definition of a scale j being greater than a scale k? More global?

The authors stated that (line 229), “The G pyramid summed over all spatial scales from eqs. 1-2 and both the light and dark channels along with the figure-ground assignments in the B cells, is the output of the grouping algorithm and provides a model of perception organization of the visual scene.” It took me a while to parse this sentence. If I understand this sentence correctly, how about saying sth. like: “The output of the model is the G pyramid summed over all spatial scales ...”?

3. Eqs. 1 and 2 define G cell activity. In the first iteration, are B0(x,y) and B0+pi(x,y) identical? If so, what is the intuition for how a G cell initially displays a figure bias? I may have missed something here. It seems that G cells may also need to integrate the simple orientation information, not just the difference between the opposing B cells.

4. The model uses an annular, donut-shape kernel for the G cells. It is impressive that such a simple proto-object with different spatial scales is able to capture the responses of many neurons. But one may also think of cases that this kernel may not work very well. What happens if an object has an elongated shape, such as a telephone pole? What happens if an object has a concave shape? It would be helpful to include a bit more discussion on the choice of the kernel and its limitation.

5. In evaluating the model performance, the authors computed linear regression fits between the cell border ownership response and the model response. It is mentioned (line 490) that the mean value of R2 goodness of fit values over all cells was 0.14. And (line 486) the R2 goodness values for the highly consistent cells ranged from 0.05-0.55, with a mean value of 0.24. My question is how to interpret the linear regression result using the R2 goodness of fit. The authors also used other measures, such as cosine similarities with statistical tests and showed more convincing agreement between the model and neural data.
