# Supplementary figures and images for: Figure-Ground Organization in Natural Scenes: Performance of a Recurrent Neural Model Compared with Neurons of Area V2
Source: eNeuro. 2019 Jun 25;6(3):ENEURO.0479-18.2019. doi: 10.1523/ENEURO.0479-18.2019 (PMC6635809; doi:10.1523/ENEURO.0479-18.2019)

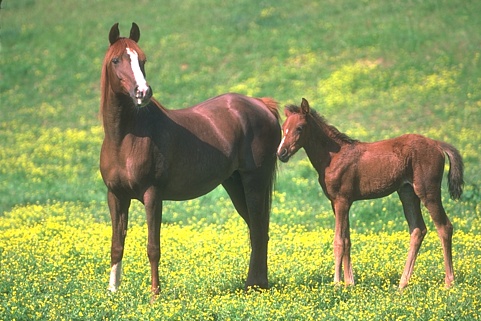

Supplement: Extended Data — Download Extended Data, ZIP file. [file sup_enu-eN-NWR-0479-18-s02.zip › FG_RNN-master/images/113016.jpg]

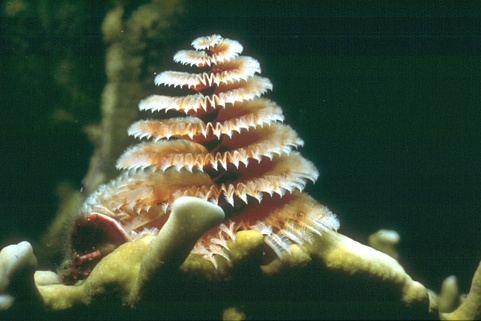

Supplement: Extended Data — Download Extended Data, ZIP file. [file sup_enu-eN-NWR-0479-18-s02.zip › FG_RNN-master/images/12074.jpg]

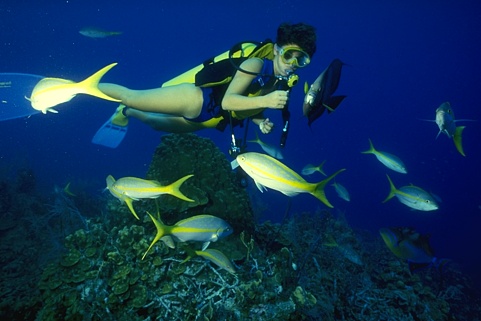

Supplement: Extended Data — Download Extended Data, ZIP file. [file sup_enu-eN-NWR-0479-18-s02.zip › FG_RNN-master/images/156079.jpg]

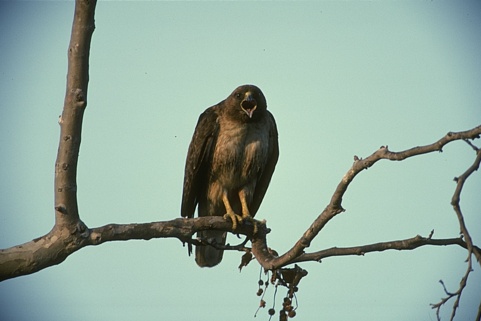

Supplement: Extended Data — Download Extended Data, ZIP file. [file sup_enu-eN-NWR-0479-18-s02.zip › FG_RNN-master/images/42049.jpg]

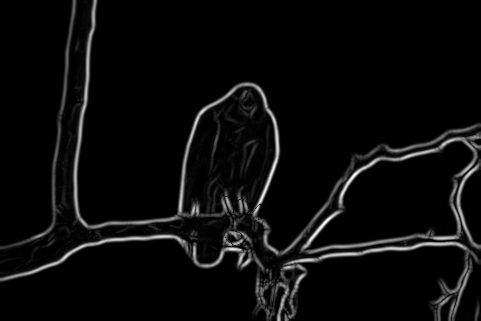

Supplement: Extended Data — Download Extended Data, ZIP file. [file sup_enu-eN-NWR-0479-18-s02.zip › FG_RNN-master/output/edge/42049.bmp]

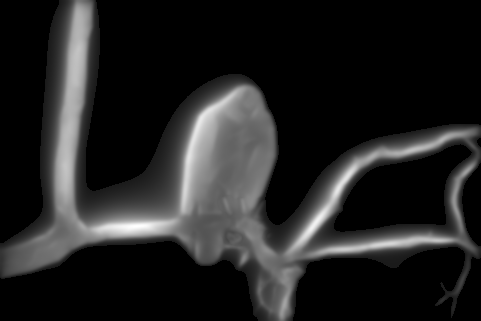

Supplement: Extended Data — Download Extended Data, ZIP file. [file sup_enu-eN-NWR-0479-18-s02.zip › FG_RNN-master/output/group/42049.bmp]

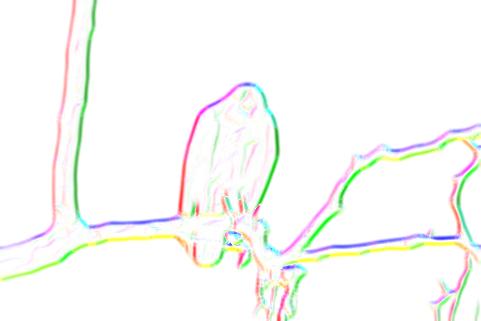

Supplement: Extended Data — Download Extended Data, ZIP file. [file sup_enu-eN-NWR-0479-18-s02.zip › FG_RNN-master/output/ori/42049.jpg]
